# Risk of Ischemic Stroke After Acute Myocardial Infarction in Patients Undergoing Coronary Artery Bypass Graft Surgery

**DOI:** 10.1038/s41598-020-60854-1

**Published:** 2020-03-02

**Authors:** André Åström, Lars Söderström, Thomas Mooe

**Affiliations:** 1 Department of Public Health and Clinical Medicine, Umeå University, Umeå, Sweden; 2 Unit of Research, Education and Development, Östersund Hospital, Östersund, Sweden; 3 Professor, Department of Public Health and Clinical Medicine, Östersund, Umeå University, Umeå, Sweden

**Keywords:** Interventional cardiology, Risk factors, Acute coronary syndromes, Vascular diseases, Preventive medicine

## Abstract

Only sparse epidemiological data are available regarding the risk of ischemic stroke (IS) after coronary artery bypass surgery (CABG). Here we aimed to describe the incidence and predictors of IS associated with CABG performed after acute myocardial infarction (AMI), as well as trends over time. We analyzed data for 248,925 unselected AMI patients. We separately analyzed groups of patients who underwent CABG early or late after the index infarction. IS incidence rates per year at risk were 15.8% (95% confidence interval, 14.5–17.1) and 10.9% (10.6–11.2), respectively, among patients with and without CABG in the early cohort, and 4.0% (3.5–4.5) and 2.3% (2.2–2.3), respectively, among patients with and without CABG in the late cohort. Predictors of post-AMI IS included prior IS, CABG, prior atrial fibrillation, prior hemorrhagic stroke, heart failure during hospitalization, older age, diabetes mellitus, and hypertension. Reduced IS risk was associated with use of statins and P2Y12 inhibitors. IS incidence markedly decreased among patients who did not undergo CABG, while no such reduction over time occurred among those who underwent CABG. This emphasizes the need to optimize modifiable risk factors and to consistently use treatments that may reduce IS risk among CABG patients.

## Introduction

Ischemic stroke (IS) is often a devastating event, carrying a high mortality rate and huge economic consequences for society^1,2^. During the early period following an acute myocardial infarction (AMI), there is an increased risk of stroke, particularly stroke of ischemic origin^3^. Although the mechanisms behind post-AMI IS are largely unknown, several risk factors have been identified, including older age, female sex, prior IS, prior diabetes mellitus, atrial fibrillation, chronic kidney disease, heart failure during hospitalization, and ST-elevation myocardial infarction (STEMI)0^4–7^. The initial increased risk of IS after AMI seems to be associated with pro-thrombotic factors, such as platelet activation, inflammation, and sympathetic activation^1,8–10^.

Patients undergoing coronary artery bypass surgery (CABG) also show an increased risk of IS, with the majority of CABG-associated strokes occurring postoperatively^11^. The most important risk factors for CABG-associated stroke include advanced age, prior cerebrovascular disease, prior carotid artery stenosis, prior peripheral vascular disease, prolonged cardiopulmonary bypass time, and postoperative atrial fibrillation^12,13^. Undergoing CABG within the acute phase after AMI has also been identified as a risk factor for post-AMI IS^2,5^. However, only limited data are available regarding the risk of IS associated with post-AMI CABG. There is a particular lack of long-term follow-up data and outcome data for patients undergoing CABG during a more stable post-AMI phase. To date, no predictors of IS in patients undergoing post-AMI CABG surgery have been conclusively identified, and additional knowledge is required to develop preventive measures.

Over the last decade, the risk of IS after AMI has decreased, likely due to changes of AMI characteristics, along with increased use of reperfusion therapies and more intense treatment with statins and antithrombotic drugs^4–6^. However, the trend over time with regards to post-AMI IS among patients undergoing CABG surgery is unknown.

In the present study, we aimed to investigate the risk of IS associated with CABG performed early (within 30 days) or late (within 31–180 days) after admission for AMI, to identify risk factors for post-AMI IS in patients undergoing CABG surgery, and to examine whether IS risk has changed over time.

## Results

**Figure 1:**
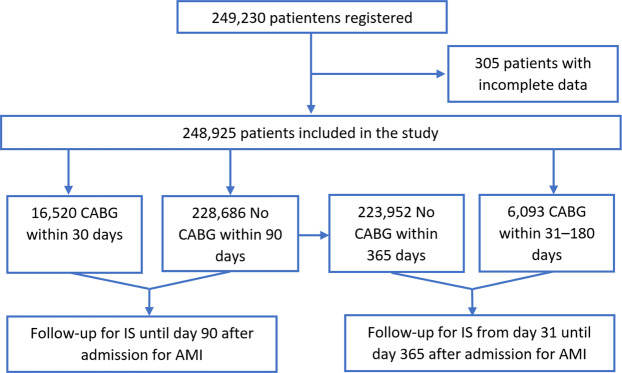
Study flow chart. CABG indicates coronary artery bypass graft. AMI indicates acute myocardial infarction. IS indicates ischemic stroke.

A total of 249,230 patients were registered in the RIKS-HIA registry for the first time during the study period. After 305 patients were excluded due to incomplete data, 248,925 patients were included in our analyses. Figure 1 shows the numbers of patients in the different study groups.

### Patient characteristics

Table 1 presents the patient characteristics at baseline. Compared to patients who did not undergo CABG, patients who underwent CABG surgery were younger and were more commonly male. The CABG cohort showed higher incidence of diabetes mellitus, and showed lower incidences of heart failure during hospitalization, prior stroke (ischemic or hemorrhagic), peripheral artery disease, and atrial fibrillation. Moreover, patients who underwent CABG less commonly received reperfusion therapy with percutaneous coronary intervention (PCI), especially in the early group (CABG within 30 days after admission for AMI).

**Table 1:** Baseline Patient Characteristics.

	No CABG within 365 days after admission % (n)	CABG within 30 days after admission % (n)	CABG within 31–180 days after admission % (n)	*P*^*a*^	*P*^*b*^	*P*^*c*^
Subjects	90.8 (223,952)	6.7 (16,520)	2.4 (6,093)			
Women	37.9 (84,931)	23.1 (3,812)	23.7 (1,444)	<0.001	<0.001	0.324
Median age, years	73	69	68	<0.001	<0.001	<0.001
STEMI/LBBB*	41.3 (90,947)	24.7 (4,019)	40.5 (2,423)	<0.001	0.214	<0.001
Hypertension	46.7 (104,678)	49.4 (8,166)	45.3 (2,758)	<0.001	0.023	<0.001
Heart failure during hospitalization^†^	34.5 (73,260)	27.4 (4,254)	31.2 (1,793)	<0.001	<0.001	<0.001
Prior atrial fibrillation	19.5 (43,659)	12.3 (2,029)	12.3 (751)	<0.001	<0.001	0.930
Peripheral artery disease	5.7 (12,655)	4.7 (771)	4.4 (268)	<0.001	<0.001	0.392
Diabetes mellitus	22.0 (49,345)	26.6 (4,393)	26.9 (1,642)	<0.001	<0.001	0.590
Prior ischemic stroke	8.9 (19,898)	5.6 (928)	5.2 (318)	<0.001	<0.001	0.244
Prior hemorrhagic stroke	1.5 (3,427)	0.8 (131)	0.9 (56)	<0.001	<0.001	0.353
PCI during hospitalization	43.0 (96,266)	11.2 (1,857)	24.2 (1,476)	<0.001	<0.001	<0.001
Thrombolysis during hospitalization	11.3 (25,353)	8.1 (1,334)	17.5 (1,068)	<0.001	<0.001	<0.001
Smoking^‡^	22.7 (45,973)	24.0 (3,724)	25.6 (1,461)	<0.001	<0.001	0.014

*P*^*a*^, significance for the comparisons of No CABG within 365 days vs. CABG within 30 days. *P*^*b*^, significance for the comparisons of No CABG within 365 days vs. CABG within 31–180 days. *P*^*c*^, significance for the comparisons of CABG within 30 days vs. CABG within 31–180 days. CABG indicates coronary artery bypass graft; LBBB, left bundle branch block; n, number of valid cases; and STEMI, ST-elevation myocardial infarction. *1.6% missing data. ^†^5.3% missing data. ^‡^9.2% missing data.

Across the study period, the proportion of patients with STEMI or left bundle branch block myocardial infarction (LBBB) decreased. The performance of reperfusion therapy with PCI increased over time. CABG was increasingly performed among patients with diabetes mellitus throughout the study period. Over time, the proportion of patients with prior atrial fibrillation increased among patients undergoing CABG, and decreased among those who did not undergo CABG, especially in the early cohort (Supplementary material, S1 Table and S2 Table).

### Therapies

Tables 2 and 3 show treatment at discharge in the early and late cohorts, stratified by time periods. Across the study period, the use of evidence-based therapies markedly increased in all groups. Patients in the early cohort who underwent CABG surgery were less commonly treated with P2Y12 inhibitors at discharge.

**Table 2:** Treatment at Discharge for Patients with Acute Myocardial Infarction, Stratified by Time Period and CABG Surgery within 30 days.

	1998–2002		2003–2007		2008–2013	
	CABG % (n)	No CABG % (n)	CABG % (n)	No CABG % (n)	CABG % (n)	No CABG % (n)
Subjects	100 (4,271)	100 (69,958)	100 (6,078)	100 (73,100)	100 (6,171)	100 (85,628)
ACE inhibitors	40.6 (1,668)	41.9 (27,253)	51.8 (3,093)	49.7 (35,784)	60.2 (3,672)	59.3 (50,605)
β-Blockers	86.1 (3,563)	78.6 (51,409)	89.8 (5,373)	83.9 (60,423)	91.2 (5,571)	85.9 (73,323)
Aspirin	88.4 (3,659)	81.3 (53,274)	92.8 (5,556)	86.0 (62,033)	95.2 (5,794)	90.4 (76,945)
Oral anticoagulants	5.7 (233)	9.1 (5,906)	3.9 (234)	6.5 (4,667)	5.5 (328)	7.1 (6,061)
P2Y12 inhibitors	12.2 (506)	14.1 (9,221)	24.8 (1,467)	61.7 (44,223)	23.2 (1,403)	81.8 (69,556)
Statins	63.4 (2,606)	42.9 (27,836)	83.9 (5,010)	68.8 (49,540)	93.4 (5,706)	82.6 (70,482)

ACE indicates angiotensin-converting enzyme; CABG, coronary artery bypass graft; and n, number of valid cases.

**Table 3:** Treatment at Discharge for Patients with Acute Myocardial Infarction, Stratified by Time Period and CABG Surgery within 31–180 days.

	1998–2002		2003–2007		2008–2013	
	CABG % (n)	No CABG % (n)	CABG % (n)	No CABG % (n)	CABG % (n)	No CABG % (n)
Subjects	100 (2,989)	100 (67,263)	100 (1,882)	100 (71,841)	100 (1,222)	100 (84,848)
ACE inhibitors	47.5 (1,384)	41.7 (26,011)	59.4 (1,113)	49.6 (35,054)	68.3 (883)	59.2 (50,075)
β-Blockers	89.2 (2,628)	78.1 (49,043)	92.0 (1,723)	83.7 (59,275)	92.0 (1,123)	85.8 (72,611)
Aspirin	89.0 (2,621)	81.0 (50,872)	93.0 (1,745)	85.9 (60,891)	95.6 (1,162)	90.4 (76,213)
Oral anticoagulants	8.2 (239)	9.1 (5,685)	5.4 (100)	6.5 (4,592)	5.5 (67)	7.1 (6,017)
P2Y12 inhibitors	12.7 (373)	14.2 (8,929)	63.2 (1,181)	61.6 (43,421)	82.9 (1,007)	81.7 (68,871)
Statins	65.1 (1,897)	42.1 (26,232)	86.6 (1,621)	68.6 (48,505)	95.0 (1,155)	82.4 (69,738)

ACE indicates angiotensin-converting enzyme; CABG, coronary artery bypass graft; and n, number of valid cases.

### Ischemic stroke incidence

**Figure 2:**
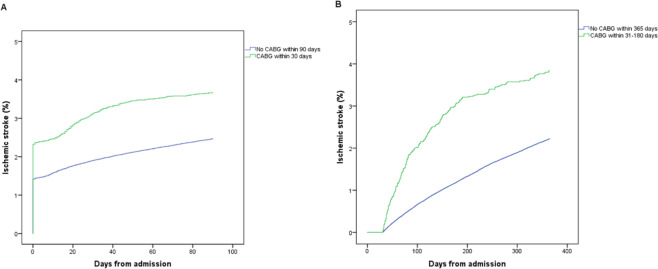
Cumulative occurrence of ischemic stroke. Cumulative occurrence of ischemic stroke (**A**) ≤90 days and (**B**) 31–365 days after admission for acute myocardial infarction, with comparison between patients who did and did not undergo coronary artery bypass graft (CABG) surgery.

Within the early cohort, the cumulative incidence of IS at 90 days was 3.7% (n = 600) for patients who underwent CABG, and 2.5% (n = 5417) for patients without CABG surgery (*P* < 0.001). Within the late cohort, the cumulative 365-day IS incidence was 3.8% (n = 227) among patients with CABG, and 2.2% (n = 4082) among patients without CABG (*P* < 0.001). In the early cohort, IS most commonly occurred during hospitalization (2.3% in CABG patients versus 1.4% in non-CABG patients). In contrast, in the late cohort, IS accumulated successively during follow-up. Outside of the time windows of CABG surgery performance, the Kaplan-Meier curves ran parallel in both the early and late cohorts (Fig. 2). IS incidence rates per year at risk were 15.8% [95% confidence interval (CI), 14.5–17.1] and 10.9% (95% CI, 10.6–11.2), respectively, for patients with and without CABG in the early cohort, and 4.0% (95% CI, 3.5–4.5) and 2.3% (95% CI, 2.2–2.3), respectively, for patients with and without CABG in the late cohort.

### Predictors

The final multivariable time-dependent Cox analysis included 226,449 patients in the early cohort and 206,416 patients in the late cohort. Consistent predictors of post-AMI IS included prior IS, CABG surgery, prior atrial fibrillation, prior hemorrhagic stroke, heart failure during hospitalization, older age, diabetes mellitus, hypertension, and use of ACE inhibitors at discharge. Female sex and STEMI/LBBB were associated with increased IS risk in the early cohort, while β-blockers at discharge and prior peripheral artery disease were associated with increased IS risk in the late cohort. Reduced IS risk was associated with PCI during hospitalization, and use of statins and P2Y12 inhibitors at discharge. Lower risk of IS was also predicted by aspirin use at discharge in the early cohort, and oral anticoagulant use at discharge in the late cohort (Tables 4 and 5). The smoking variable was excluded due to missing data.

**Table 4:** Multivariable Time-Dependent Cox Regression Analysis of Predictors for Ischemic Stroke ≤90 Days After Acute Myocardial Infarction.

	HR	95% CI	*P* Value
Age	1.02	1.02–1.03	<0.001
Women	1.14	1.08–1.21	<0.001
STEMI/LBBB	1.19	1.12–1.25	<0.001
CABG within 30 days	1.53	1.30–1.79	<0.001
Prior ischemic stroke	2.63	2.46–2.80	<0.001
Prior hemorrhagic stroke	1.55	1.33–1.81	<0.001
Prior atrial fibrillation	1.56	1.47–1.66	<0.001
Prior diabetes mellitus	1.19	1.12–1.26	<0.001
Hypertension	1.11	1.05–1.18	0.003
Heart failure during hospitalization	1.24	1.17–1.32	<0.001
ACE inhibitors at discharge	1.09	1.03–1.15	0.003
Aspirin at discharge	0.75	0.70–0.80	<0.001
P2Y12 inhibitors at discharge	0.81	0.76–0.87	<0.001
Statins at discharge	0.87	0.81–0.92	<0.001
PCI during hospitalization	0.77	0.71–0.84	<0.001

ACE indicates angiotensin-converting enzyme; CABG, coronary artery bypass graft, included as a time-dependent variable; CI, confidence interval; HR, hazard ratio; LBBB, left bundle branch block; PCI, percutaneous coronary intervention; and STEMI, ST-elevation myocardial infarction.

**Table 5:** Multivariable Time-Dependent Cox Regression Analysis of Predictors for Ischemic Stroke 31–365 Days After Acute Myocardial Infarction.

	HR	95% CI	*P* Value
Age	1.04	1.04–1.04	<0.001
Women	1.03	0.97–1.10	0.335
CABG within 31–180 days	2.80	2.45–3.20	<0.001
Prior ischemic stroke	2.60	2.41–2.80	<0.001
Prior hemorrhagic stroke	1.46	1.21–1.76	<0.001
Prior atrial fibrillation	1.88	1.76–2.02	<0.001
Prior diabetes mellitus	1.34	1.25–1.43	<0.001
Prior peripheral artery disease	1.16	1.04–1.29	0.009
Hypertension	1.15	1.08–1.23	<0.001
Heart failure during hospitalization	1.13	1.06–1.21	0.001
ACE inhibitors at discharge	1.10	1.03–1.17	0.003
β-Blockers at discharge	1.12	1.03–1.22	0.009
P2Y12 inhibitors at discharge	0.90	0.84–0.97	0.009
Statins at discharge	0.79	0.73–0.84	<0.001
Oral anticoagulants at discharge	0.78	0.70–0.87	<0.001
PCI during hospitalization	0.72	0.66–0.79	<0.001

ACE indicates angiotensin-converting enzyme; CABG, coronary artery bypass graft, included as a time-dependent variable; CI, confidence interval; HR, hazard ratio; LBBB, left bundle branch block; PCI, percutaneous coronary intervention; and STEMI, ST-elevation myocardial infarction.

### Trends over time

**Figure 3:**
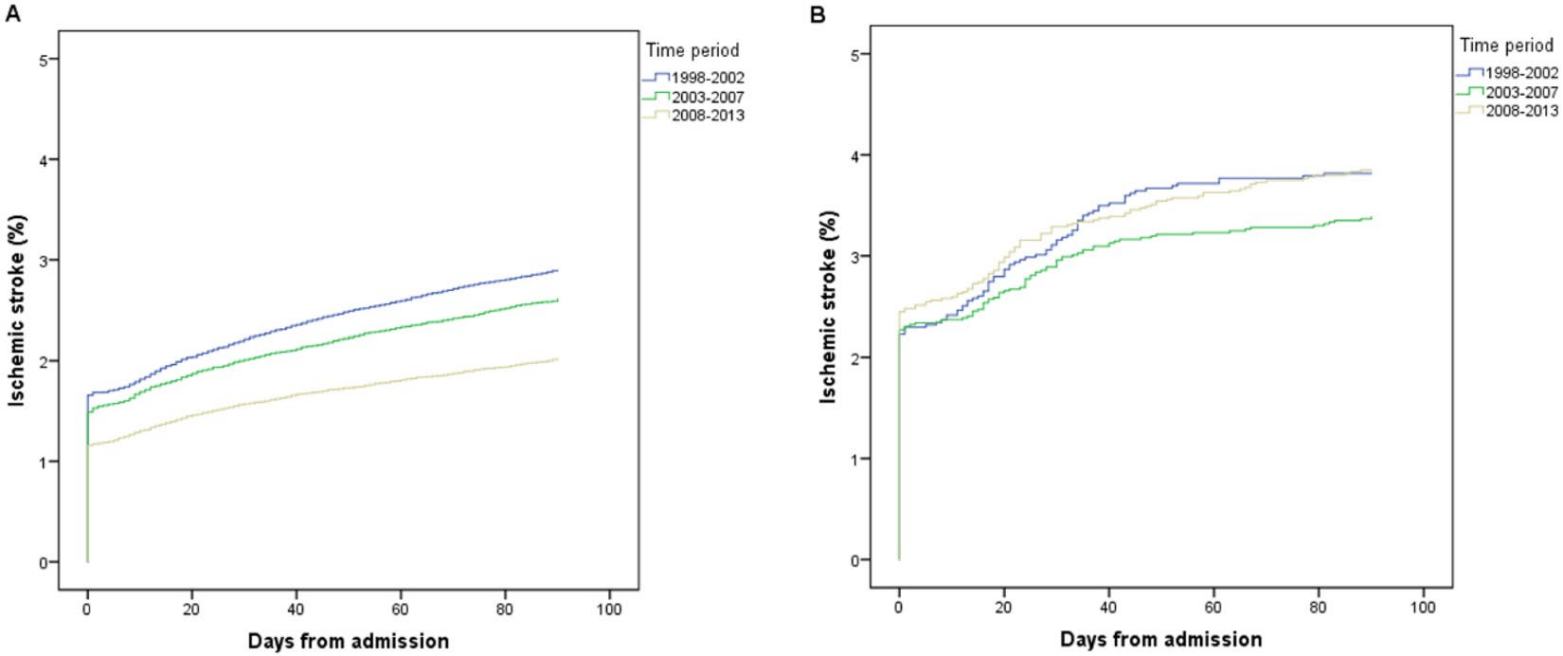
Cumulative occurrence of ischemic stroke within 90 days after admission for acute myocardial infarction. Patients who did not undergo coronary artery bypass graft (CABG) surgery within 90 days after admission (**A**) compared with those who underwent CABG surgery within 30 days (**B**), stratified by time period.

**Figure 4:**
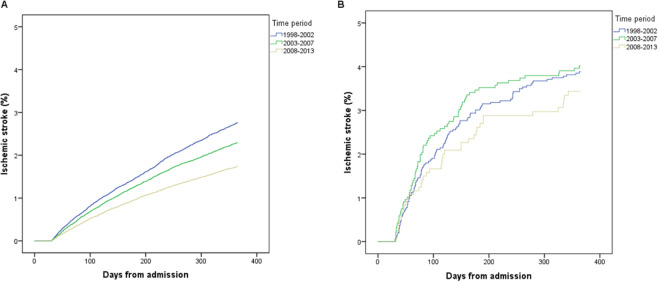
Cumulative occurrence of ischemic stroke within 31–365 days after admission for acute myocardial infarction. Patients who did not undergo coronary artery bypass graft (CABG) surgery within 365 days after admission (**A**) compared with those who underwent CABG surgery within 31–180 days (**B**), stratified by time period.

The cumulative incidence of post-AMI IS decreased markedly over the study period among patients who did not undergo CABG surgery (*P* < 0.001 for both the early and late cohorts). No such reduction over time was observed among patients who underwent CABG surgery, neither within 30 days after admission for AMI (*P* = 0.35) nor within 31–180 (*P* = 0.69) days after admission for AMI (Figs. 3 and 4).

## Discussion

Our present results verified that IS risk was increased when CABG was performed early after AMI. Moreover, IS risk was also increased when CABG was performed later after AMI, although the incidence rate was markedly lower with late CABG compared to early CABG. Importantly, a large proportion of IS in the early cohort occurred during hospitalization. Thus, preventive measures in this cohort need to be implemented in-hospital.

Our analysis confirmed several previously identified risk factors for post-CABG IS, including older age, prior cerebrovascular disease, and atrial fibrillation. We also found that several other potentially modifiable conditions were associated with increased IS risk, including prior diabetes, hypertension, and heart failure during hospitalization. Furthermore, reduced IS risk was associated with treatment with statins, P2Y12 inhibitors, aspirin (only in the early cohort), and oral anticoagulants (only in the late cohort). Notably, over the study period, IS risk decreased among patients who did not undergo CABG, but this decrease was not observed among patients who underwent CABG (in either the early or late groups).

## Methods

Any cut-off regarding time for CABG and follow-up after AMI is arbitrary. In the early cohort we included CABG within 30 days of AMI. Because of different pro-thrombotic mechanisms, the IS risk is markedly higher early after AMI. Surgery during this period may be synergistic regarding IS risk. The 90-day follow-up window was chosen to cover early complications and was applied to both groups (CABG and no CABG) to compare IS occurrence between groups. We added a late cohort with surgery within 31–180 days after admission for AMI to illustrate IS risk during a more stable phase with less inflammation and other pro-thrombotic mechanisms. We chose a follow-up until one year after AMI and compared the occurrence of IS during days 31–365 in both groups (CABG and no CABG).

The exact date of IS during the hospital stay was not available in the database. Since an acute IS is a fairly strong contraindication rather than an indication for CABG it seems fair to assume that the vast majority of IS occurred during or after surgery. Thus, some patients have probably been excluded from surgery during follow-up because of an IS but the impact on the cumulative incidence of IS in the large non-CABG group would be very small.

CABG after AMI is a time-dependent variable which we accounted for in the Cox analyses.

### Ischemic stroke incidence

Our large dataset enabled us to make precise estimates of IS risk with and without CABG surgery after AMI. Additionally, the fairly unselected study population makes it possible to generalize our present results to other general Caucasian AMI populations. Compared to our present findings, previous studies investigating CABG-associated stroke in the absence of prior AMI have reported a lower IS risk, ranging from 1.1–2.5% in studies with 30 days of follow-up^12^.

Few prior studies have investigated IS risk associated with CABG performed early versus late following AMI^2,14–17^. Moreover, it is difficult to compare our results with previous findings as prior studies lack comparison with non-CABG groups; have small study populations; and differ with regards to patient selection, outcome definitions, and definitions of early versus late CABG. In general, early post-AMI CABG has been associated with higher IS risk compared to late post-AMI CABG. However, this is expected since the risk of IS rapidly decreases over time following an AMI^1^. Compared to the non-CABG groups, the early and late CABG groups were associated with 4.9% and 1.7% higher IS incidence rates, respectively.

**Figure 5:**
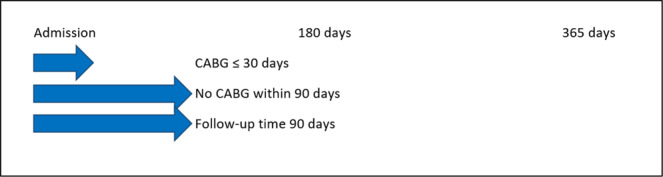
Early cohort. Coronary artery bypass graft (CABG) within 30 days after admission for acute myocardial infarction versus no CABG within 90 days. Both groups had follow-up for ischemic stroke until 90 days from admission.

**Figure 6:**
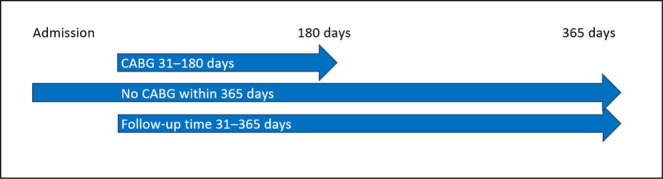
Late cohort. Coronary artery bypass graft (CABG) within 31–180 days after admission for acute myocardial infarction versus no CABG within 365 days. Both groups had follow-up for ischemic stroke between 31–365 days from admission.

Since a specific date was not available for IS during the hospital stay, IS events in-hospital accumulate on day one in the Kaplan-Meier analysis (Figs. 4–6).

The Kaplan-Meier curves for the CABG and non-CABG groups separated during the period around the time of surgery (until 30 days after AMI in the early cohort; between 31–180 days in the late cohort), but ran parallel afterwards. This is an important finding since it indicates that the baseline IS risk was similar between the CABG and non-CABG cohorts despite differences in patient characteristics resulting from the selection of patients for surgery. Thus, the increased IS risk observed in the CABG groups may mainly be explained by the surgery itself. Prior findings support that the postoperative risk of stroke peaks shortly after surgery, and decreases to normal hazard level within approximately a week^11,12^.

### Predictors

The most important predictors of IS were prior IS, CABG surgery, prior hemorrhagic stroke, and prior atrial fibrillation. We also identified several potentially modifiable predictors of increased IS risk, including prior diabetes, hypertension, and heart failure during hospitalization. The possibility of optimizing the treatment of these conditions represents the potential to decrease CABG-associated IS risk, at least in cases where surgery can be delayed. We found that several treatments were associated with decreased IS risk, including aspirin, oral anticoagulants, P2Y12 inhibitors, and statins, which should be used as consistently as possible according to guidelines. Our results showed particularly low use of P2Y12 inhibitors. Antiplatelet therapy with P2Y12 inhibitors has previously been associated with lower IS risk^5–7^, and clinical trials, scientific statement from the American Heart Association and guidelines from the European Association For Cardio-Thoracic Surgery support increased use of P2Y12 inhibitors^18–22^.

The finding that aspirin use at discharge predicted lower risk of IS in the early cohort, while oral anticoagulant use at discharge predicted lower risk in the late cohort is probably reflecting different mechanisms involved in the IS pathogenesis depending on the time from AMI. This is consistent with findings from randomized studies. Aspirin effectively reduced IS early after AMI^23^, while oral anticoagulant treatment (warfarin) was effective during the more stable phase after AMI^24^.

We also found that PCI was associated with lower IS risk, which is consistent with previous reports^25^. PCI performed early during the course of an AMI may reduce the extent of the infarction, thereby decreasing IS risk. Moreover, preserved myocardial function is beneficial in the event of later CABG surgery, potentially reducing the CABG-associated IS risk.

Our data indicated that use of ACE inhibitors at discharge was a predictor for IS; however, the lower limit of the 95% confidence interval was close to 1. Due to the observational nature of the present study, it is possible that this finding is an example of confounding by indication^5^. The increased risk of IS associated with β-blocker use at discharge within the late cohort may be similarly explained.

### Time trends

Over the study period, we observed a marked reduction of IS risk over time in the non-CABG cohorts, but not among patients undergoing CABG. This difference in trends between the CABG and non-CABG cohorts may have been influenced by the following observations in the CABG group: low use of P2Y12 inhibitors, increased prevalence of diabetes mellitus and atrial fibrillation, and smaller decrease over time of congestive heart failure during hospitalization (Table 2, Supplementary material, Table 1). The need for improved medical treatment among patients undergoing CABG has been previously described^26^. Furthermore, it may be possible to improve intraoperative and perioperative stroke preventive measures, but this is beyond the scope of the present study.

### Limitations

One limitation of the present study was that we lacked access to any intraoperative or perioperative data from the surgical procedure. Different surgical approaches reportedly have varying influences on the risk for postoperative IS^12,27^. Prolonged cardiopulmonary bypass time is consistently identified as a predictor of postoperative stroke after CABG; therefore, it would have been of interest to include procedural data in our analyses.

We also lacked access to information regarding treatment at discharge after rehospitalization for CABG. This limitation mainly affected analysis of the late cohort, as many patients in the late cohort were rehospitalized for surgery. Based on the routine treatment protocol during the study period, most patients would not have been treated with P2Y12 inhibitors at discharge after CABG surgery.

Notably, the follow-up windows for IS (90 days and 31–365 days after admission for AMI) and the 30-day cut-off for early versus late CABG were arbitrarily selected as discussed above. This limitation is common to all studies in this field since there is no established cut-off point for these different measurements. However, the analysis of Kaplan-Meier curves enables the assessment of IS risk over time independently of the starting point.

Finally, as this was an observational cohort study based on registry data, there is an inherent risk that confounders will not be taken into account because they were not recorded as variables in the database.

## Conclusions

Overall, our present results showed that IS incidence after an AMI was higher among patients who underwent CABG compared to in non-CABG patients, regardless of whether CABG was performed early or late after AMI admission. The IS incidence rate was greater in the early cohort than in the late cohort, and this difference was greater among patients who underwent CABG compared to in the non-CABG group.

The most important predictors of post-AMI IS were prior ischemic stroke and CABG surgery. We identified several potentially modifiable factors as being associated with higher IS risk, including diabetes mellitus, prior atrial fibrillation, hypertension, and heart failure during hospitalization. Moreover, reduced IS risk was predicted by treatment with PCI during hospitalization and by treatment with aspirin, oral anticoagulants, P2Y12 inhibitors, and statins after discharge.

Over the study period, IS incidence markedly decreased among non-CABG patients, but not among the patients who underwent CABG. Our findings underscore the need to optimize modifiable risk factors, as well as to use treatments associated with decreased IS risk as consistently as possible.

## Methods

This study included patients who were registered for the first time in the Swedish Register of Information and Knowledge about Swedish Heart Intensive Care Admissions (RIKS-HIA) between January 1998 and December 2013. In 1995, RIKS-HIA was first instituted as a national registry, and included 19 hospitals. Since then, it has successively grown. At the start of our study period (1998), RIKS-HIA included 58 of 81 Swedish hospitals. Since 2008, it has included all 74 hospitals in Sweden with coronary care units^28^. For each patient, over 100 variables are registered in RIKS-HIA at admission, during hospitalization, and at discharge. Annual examinations of the validity of the data entered in the register reveal 94–97% conformity between RIKS-HIA data and patient records^28,29^.

For the first several years of the study period, the World Health Organization’s definition^30^ was used as the criteria for AMI. In 2001, RIKS-HIA began to define AMI using the definition formulated by the European Society of Cardiology/American College of Cardiology/American Heart Association^31,32^. To determine the incidence of IS within 365 days of admission for AMI, we merged the RIKS-HIA database with the Swedish National Patient Register (NPR), which includes admission and discharge dates, and diagnoses at discharge for all hospital stays in Sweden. The International Classification of Diseases 9th Revision (433 and 434) and 10th Revision (I63 and I64) codes for cerebral infarction were used. Since 2000, there has been only a low frequency of missing primary diagnoses in NPR (0.5–0.9% in somatic care). A diagnosis of stroke or transient ischemic attack (TIA) in NPR has a positive predictive value of 98.6%. Validations studies show that 84.2–98% of stroke events are identified through the NPR^33^.

CABG surgery within 30 days after admission for AMI was defined as early, representing the acute post-AMI phase. Follow-up for IS in this early CABG group was from admission until 90 days, and this cohort was compared with patients who did not undergo CABG within 90 days of admission for AMI, Fig. 5. CABG surgery within 31–180 days after admission for AMI was defined as late, representing the later and more stable post-AMI phase. Follow-up for IS in this late CABG group was from day 31 until day 365 after admission for AMI, and this cohort was compared with patients who did not undergo CABG within 365 days after admission for AMI, Fig. 6.

The follow-up periods were chosen to include any potential pro-thrombotic effects of CABG surgery (e.g., inflammation and platelet activation) and to cover both the early period and the later and more stable period.

During their hospital stay, all patients were informed of their inclusion in the RIKS-HIA registry. The registry has been approved by the National Board of Health and Welfare and the Swedish Data Inspection Board. This study was performed in accordance with the Declaration of Helsinki. The Regional Ethics Committee in Stockholm approved the merging of the registries, and waived the requirement for written consent.

### Statistical analysis

Baseline characteristics were compiled for three groups: CABG within 30 days after admission for AMI, CABG within 31–180 days after admission for AMI, and no CABG surgery within 365 days after admission for AMI. The group No CABG within 90 days after admission for AMI was not separately presented because the characteristics did not differ compared to the group No CABG within 365 days after admission for AMI. Between-group comparisons were performed using the chi-square test for categorical variables, and the Mann-Whitney U-test for continuous data. Kaplan-Meier analyses were performed to estimate the cumulative incidence of IS in the different patient groups. Between-group comparisons were made using the log-rank test. We also calculated incidence rates per year at risk to enable comparisons of IS rates at different periods of time within one year after AMI. To examine trends over time, the study period was divided into three time periods: 1998–2002, 2003–2007, and 2008–2013.

Cox proportional hazards regression analyses with CABG as a time dependent co-variate were performed to assess univariable and multivariable predictors of IS. The multivariable models included variables having a *P* value of <0.1 in the univariable analyses. We performed stepwise reduction of the models by manually excluding the least significant variables until only significant variables remained. Gender was retained in the models regardless of significance. Only up to 10% missing data was accepted in the final model. A *P* value of <0.05 was considered significant. Time-dependent Cox proportional hazards regression analyses were performed using SAS 9.4 software (SAS Institute Inc., Cary, NC, USA). All other statistical analyses were performed using SPSS 23.0 software (IBM Corp., Armonk, NY, USA).

## Supplementary information

Supplementary Data.
