# Supplementary material for: Risk of Ischemic Stroke After Acute Myocardial Infarction in Patients Undergoing Coronary Artery Bypass Graft Surgery
Source: Sci Rep. 2020 Mar 2;10:3831. doi: 10.1038/s41598-020-60854-1 (PMC7052208; doi:10.1038/s41598-020-60854-1)
Supplement: Supplementary file 1 — Supplementary Data. [file 41598_2020_60854_MOESM1_ESM.pdf]

Supplementary Material

Risk of Ischemic Stroke After Acute Myocardial Infarction in Patients Undergoing Coronary Artery Bypass Graft Surgery

André Åström[1], Lars Söderström[2], and Thomas Mooe[3*]

[1]MD, Department of Public Health and Clinical Medicine, Umeå University, Umeå, Sweden

[2]MSc, Unit of Research, Education and Development, Östersund Hospital, Östersund, Sweden

[3]MD, Professor, Department of Public Health and Clinical Medicine, Östersund, Umeå University, Umeå, Sweden

[*]thomas.mooe@umu.se

Supplementary tables

S1 Table. Patient characteristics stratified by time period and CABG surgery within 30 days

	1998–2002		2003–2007		2008–2013	
	CABG % (n)	No CABG % (n)	CABG % (n)	No CABG % (n)	CABG % (n)	No CABG % (n)
Subjects	100 (4,271)	100 (69,958)	100 (6,078)	100 (73,100)	100 (6,171)	100 (85,628)
Women	24.9 (1,062)	37.6 (26,299)	23.0 (1,396)	38.3 (27,966)	21.9 (1,354)	37.2 (31,838)
STEMI/LBBB	32.5 (1,345)	47.1 (31,841)	26.3 (1,578)	41.7 (30,127)	17.9 (1,096)	36.4 (31,031)
PCI during hospitalization	5.2 (224)	14.4 (10,072)	11.5 (700)	46.1 (33,722)	15.1 (933)	62.8 (53,790)
Prior ischemic stroke	5.4 (230)	8.9 (6,196)	5.7 (348)	9.4 (6,864)	5.7 (350)	8.3 (7,066)
Heart failure during hospitalization	32.2 (1,298)	44.3 (29,136)	28.9 (1,606)	36.0 (24,632)	22.7 (1,350)	25.3 (20,921)
Prior atrial fibrillation	10.9 (465)	20.1 (14,056)	12.0 (731)	19.9 (14,564)	13.5 (833)	18.3 (15,636)
Diabetes Mellitus	24.3 (1,036)	22.1 (15,469)	26.0 (1,579)	22.2 (16,194)	28.8 (1,778)	22.1 (18,914)
Prior hemorrhagic stroke	0.5 (23)	1.5 (1,017)	0.7 (43)	1.5 (1,133)	1.1 (65)	1.5 (1,318)
Hypertension	42.6 (1,820)	37.7 (26,364)	48.3 (2,937)	46.0 (33,622)	55.2 (3,409)	54.6 (46,761)

ACE indicates angiotensin-converting enzyme; CABG, coronary artery bypass graft; LBBB, left bundle branch block; n, number of valid cases; PCI, percutaneous coronary intervention; and STEMI, ST-elevation myocardial infarction.

S2 Table. Patient characteristics stratified by time period and CABG surgery within 31-180 days

	1998–2002		2003–2007		2008–2013	
	CABG % (n)	No CABG % (n)	CABG % (n)	No CABG % (n)	CABG % (n)	No CABG % (n)
Subjects	100 (2,989)	100 (67,263)	100 (1,882)	100 (71,841)	100 (1,222)	100 (84,848)
Women	23.9 (714)	38.1 (25,649)	24.4 (459)	38.5 (27,639)	22.2 (271)	37.3 (31,643)
STEMI/LBBB	39.8 (1,158)	47.2 (30,681)	40.4 (754)	41.7 (29,584)	42.1 (511)	36.3 (30,682)
PCI during hospitalization	8.8 (262)	14.6 (9,796)	29.7 (559)	46.2 (33,213)	53.6 (655)	62.8 (53,257)
Prior ischemic stroke	5.2 (156)	9.0 (6,080)	5.0 (959)	9.5 (6,792)	5.5 (67)	8.3 (7,026)
Heart failure during hospitalization	32.1 (903)	44.8 (28,303)	33.3 (583)	36.0 (24,238)	26.1 (307)	25.2 (20,719)
Prior atrial fibrillation	11.5 (343)	20.4 (13,721)	13.4 (253)	20.0 (14,395)	12.7 (155)	18.3 (15,543)
Diabetes Mellitus	26.1 (781)	22.0 (14,808)	26.7 (503)	22.1 (15,846)	29.3 (358)	22.0 (18,691)
Prior hemorrhagic stroke	0.7 (22)	1.5 (1,002)	0.9 (17)	1.6 (1,117)	1.4 (17)	1.5 (1,308)
Hypertension	39.5 (1,182)	37.7 (25,355)	48.0 (903)	45.9 (32,997)	55.1 (673)	54.6 (46,326)

ACE indicates angiotensin-converting enzyme; CABG, coronary artery bypass graft; LBBB, left bundle branch block; n, number of valid cases; PCI, percutaneous coronary intervention; and STEMI, ST-elevation myocardial infarction.
